# ArYSL: Arabic Yemeni sign language dataset

**DOI:** 10.1016/j.dib.2025.111996

**Published:** 2025-08-19

**Authors:** Mogeeb A․ A․ Mosleh, Rehab A.A. Mohammed, Ahme A.A. Mohammed, Abdu H. Gumaei

**Affiliations:** a Engineering and Computing Faculty – University of Science and Technology, Aden, Yemen; b Software Engineering Department – Faculty of Engineering and Information Technology – Taiz University Taiz 6308, Yemen

**Keywords:** Sign language, Arabic sign language (ARSL), Arabic sign dataset, Arabic deaf community

## Abstract

Recognition of Arabic Sign Language (ARSL) remains a significant challenge due to the lack of extensive datasets, particularly those that reflect hand signs in real-life situations. The ArYSL Version 2 dataset was proposed to address such limitations by creating a nuanced Arabic Yemeni Sign Language to be used for Arabic sign translation tasks. The ArYSL Version 2 dataset is an expanded edition of Version 1, comprising 32 Arabic sign classes and 35,900 labeled RGB images collected from 35 participants of diverse ages and genders. In addition, ArYSL Version 2 dataset is enhanced with a curated dictionary of 357 Arabic words incorporating synonyms, dialectal variations and common misspellings. It is a suitable resource to utilize deep learning and fuzzy logic methods for bidirectional translation in both image-to-text and text-to-image tasks. The primary contribution of this work is the development and release of a large dynamic word-based, fully-labeled dataset of Arabic Yemeni Sign Language ArYSL Version 2 accompanied by details of Arabic data dictionaries. It is freely available online for the research community and can be publicly accessed at: https://doi.org/10.6084/m9.figshare.26114395.v1

Specifications Table

**Table:** 

Subject	Computer Sciences, Computer Vision, Deep Learning, Fuzzy Logic
Specific subject area	Sign Language Recognition, Bidirectional Sign-to-Text and Text-to-Sign Translation.
Type of data	Labeled RGB images (JPEG/PNG) format; Metadata files (CSV, NPY) for metadata and dictionary; Python scripts used for acquisition, preprocessing, quality control, sign translation Demo; documentation files.
Data collection	The ArYSL Version 2 database consists of nearly 35,900 annotated RGB frames with 32 classes of Arabic signs. Datasets are grouped into labeled directories containing metadata CSVs in addition to NPY files and the 357-word Arabic dictionary to enable semantic mapping and models training. • Hardware: Data was captured using consumer-grade devices including iPhone 6s, Samsung Galaxy A52, Samsung Galaxy Note 1, and HP ZBook laptop. • Tools and Software: Python 3.10+ was used, along with key libraries such as OpenCV, NumPy, Pandas, FuzzyWuzzy, and TensorFlow/Keras. To support reproducibility, both a requirements.txt and an environment.yml file—detailing the complete software environment and library versions—are included in the dataset repository. • Capture Conditions: The dataset was curated to simulate real-world usage conditions. Backgrounds were uncontrolled but partially standardized in some sessions using black fabric or curtains. • Gesture Recording: Participants were instructed to perform each sign multiple times with different variations. minimum of 680 images were captured for each class. • Dataset Structure: Each sign class is stored in a separate directory (e.g., /doctor/, /how/, /wait/), containing at least 680 images per class. • Data Quality Control: Manual and automated filtering was performed to exclude corrupted, blurred, or incorrectly labeled images. Image quality was assessed using Laplacian variance and histogram-based checks.
Data source location	Multiple regions in Taiz City, Yemen, across different schools and one university the data collection process coordinated by Software Engineering Department – Taiz University – Taiz City – 3086, Yemen
Data accessibility	Repository name: ArYSL: Arabic Yemeni Sign Language [1] • Data identification number (DOI): https://doi.org/10.6084/m9.figshare.26114395.v1. • Direct URL to data: https://figshare.com/articles/dataset/_b_Yemeni_sign_Language_dataset_b_/26114395. • Instructions for accessing these data: Publicly available for academic and educational purposes. No login required to download.
Related research article	Mosleh, M. A. A., & Gumaei, A. H. (2024). An Efficient Bidirectional Android Translation Prototype for Yemeni Sign Language Using Fuzzy Logic and CNN Transfer Learning Models. DOI: 10.1109/ACCESS.2024.3512455 Publisher: IEEE Access. URL: https://ieeexplore.ieee.org/abstract/document/10778485 [2].

## 1 Value of the Data

• It is the first open dataset of Arabic Yemeni Sign Language (ArYSL) at a large scale and is intended to facilitate the development of accessible, region-localized sign language technology for Arabic-speaking people.
• The dataset is freely available to be used for academic and research purposes, and is legally restricted for non-commercial applications.
• ArYSL Version 2 is a dataset of 35,900 RGB pictures for 32 culturally interested stationary sign categories, taken in semi-uncontrolled real-life setups.
• It supports two-way translation between written Arabic and Yemeni Sign Language through a structured dictionary of 357 Arabic words, incorporating synonyms, nuanced forms, phonetic spelling variations, and common errors.
• Researchers and interested experts can develop and explore machine learning systems using this dataset for bidirectional sign translators, including use cases such as:
  ○ Developing baseline CNN models for sign classification
  ○ Integrating fuzzy matching for Arabic NLP pipelines in text-to-sign translation
  ○ Building mobile and web applications for deaf education and assistive communication.
• The ArYSL Version 2 dataset has been evaluated using several CNN models—such as MobileNetV2, ResNet152, DenseNet121, InceptionV3, Xception, and VGG16—in the aforementioned research activities, demonstrating its effectiveness in real-time deployable sign recognition systems [[2], [3], [4]].

## 2 Background

Arabic Sign Language recognition systems had limited access to any large publicly available datasets, particularly those with the diversity and subtlety of regional dialects. Existing resources are often limited by small vocabularies, constrained environments [5], alphabet-based datasets [6,7], or standardization approaches that fail to represent real-world application. To overcome these difficulties, the ArYSL Version 2 dataset was created to provide a large variety of nearly 35,900 sign images. This data article describes the most complete ArYSL Version 2 dataset that builds on previous releases by expanding the number of sign classes and enhancing preprocessing procedures.

## 3 Data Description

The Arabic Yemeni Sign Language (ArYSL) Version 2 dataset is a high-resolution, curated dataset that can support research toward the automatic bidirectional recognition and translation of the Arabic Sign Language. This new version presents a major improvement of the previous one ArYS Version 1 dataset [8], by expanding it in the coverage of classes, number of samples, and the inclusion of a structured lexical mapping resource. The ArYSL Version 1 of the dataset included 14 sign classes as part of preliminary research; however, detailed participant documentation was not recorded at that stage. The images of ArYSL Version 1 were re-verified and re-annotated under the standardized protocols established for Version 2 to ensure consistency in labeling, image quality, and metadata structure. After validation, approximately 11,637 sign images from ArYSL Version 1 were retained and integrated with the newly collected data from this study to construct the final comprehensive dataset, referred to as ArYSL Version 2. In contrast, this study involved 35 participants who contributed a total of 24,246 new and distinct sign images across 18 sign classes. These were integrated with the existing images from 14 additional classes in the original ArYSL Version 1 dataset. As a result, the final ArYSL Version 2 dataset comprises approximately 35,900 annotated images covering 32 sign classes. Based on the documented records from ArYSL Version 2, this equates to an average of approximately 693 sign images per participant.

This dataset was carefully constructed to support machine learning training, validation, and evaluation of sign language interpretation and follows semantic definitions established in the Unified Yemeni Sign Language Dictionary, which preserves linguistic fidelity and consistency in the classes. Data dictionary was designed in a systematic fashion to enable the translation of signs to text. The dictionary serves as an essential layer to map Arabic text into its associated sign language gesture. It had a formal procedure that involved source reference verification, information of keyword, semantic mapping of signs with words in Arabic, and extensive data normalization of information to maintain uniformity and interoperability within the translation system. The ArYSL Version 2 dataset is organized into 32 distinct sign classes, each representing culturally relevant words commonly expressed through gestures in the daily communication practices of Yemeni society. Each of these classes is stored in its own directory, avoiding any overlap between subsets of at least 680 images per class, with some exceptions based on frequency and significance of the sign in the real world to create semantic balance. The sign images are saved in standard JPG or PNG formats with a resolution of 224×224 pixels per image. A structured lexical dictionary comprising 357 Arabic words with their various diacritics, spelling variants, and common misspelling encoded in UTF-8, and mapped to corresponding sign classes and images of gestures. The mapping results were compiled into a CSV file, with structured columns included Sign Class, Arabic word, nuanced variant and synonym, and common mistakes typing. Table 1, illustrates the ArYSL Version 2 dataset summary.

**Table 1:** ArYSL version 2 dataset summary.

Attribute	Description
Total Images	∼35,900
Gesture Classes	32
Image Format	JPEG or PNG
Resolution	224×224 pixels
Total Size	∼1.59 GB
Metadata Files	.csv (labels, counts), .npy (pre-processed tensors)
Text Mapping	357 Arabic words to gesture classes

This dataset is intended to be well-suited for a variety of applications, including:

• Deep learning-based gesture classification for Real-time translation and interpretation.
• Assistive communication tools for individuals who are deaf or hard-of-hearing.
• NLP-integrated gesture synthesis and parsing.

## 4 Experimental Design, Materials and Methods

This section describes the procedures, tools, and methodologies used in the creation of ArYSL Version 2 dataset. This Dataset was constructed mainly to improve the sign recognition, and support research on automatic bidirectional Arabic Sign Language translation.

### 4.1 Workflow overview

**Fig. 1:**
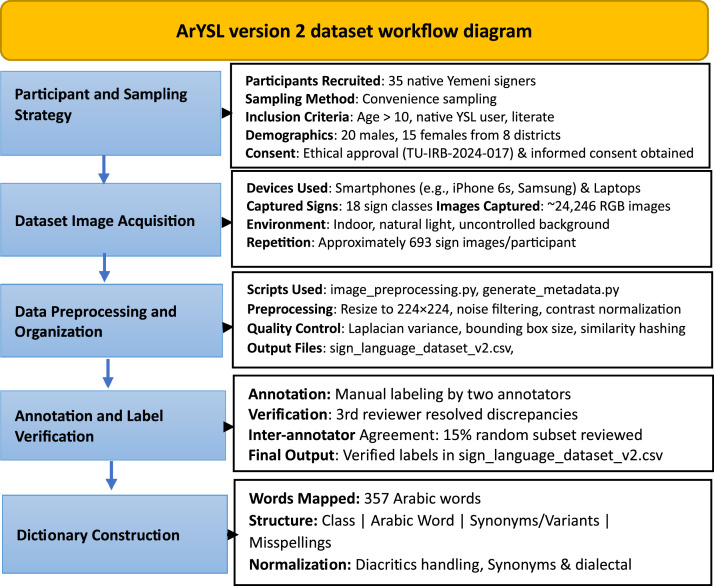
Overview of the ArYSL version 2 dataset workflow.

To enhance transparency and reproducibility, a comprehensive workflow diagram is presented in Fig. 1, illustrating the end-to-end process of constructing the ArYSL Version 2 dataset.

The pipeline starts with the recruitment of 35 native Yemeni signers, followed by image acquisition phase using various mobile and laptop devices in semi-natural environments. Each captured image underwent preprocessing steps including noise filtering, contrast normalization, and resizing. Quality control procedures were applied to ensure consistency by utilizing specific metrics thresholds included Laplacian variance, histogram similarity, and bounding box to exclude the low-quality or redundant image samples. Subsequently, validated images are next placed in a defined folder structure with metadata files created in a script-based way. Two trained annotators with an independent expert reviewer conducted the verification of annotations to provide semantic fidelity. Finally, a structured multilingual text-to-sign dictionary of 357 Arabic words is constructed mapping each sign classes based on linguistic principles and typo errors. This flexible pipeline had an impressive level of reproducibility to form a basis of real-time bidirectional sign language translation systems. This workflow summarizes the full process of data acquisition, preprocessing, quality control, metadata generation, and text-to-sign dictionary construction. Each phase is supported by custom Python scripts provided with the ArYSl Version 2 dataset repository. Finally, a comprehensive README file is included in the dataset repository, offering a detailed overview of the dataset structure, usage instructions, and example Python scripts for data loading, preprocessing, CNN training and validation, as well as integration with the text-to-sign mapping dictionary.

### 4.2 Participants and sampling strategy

**Fig. 2:**
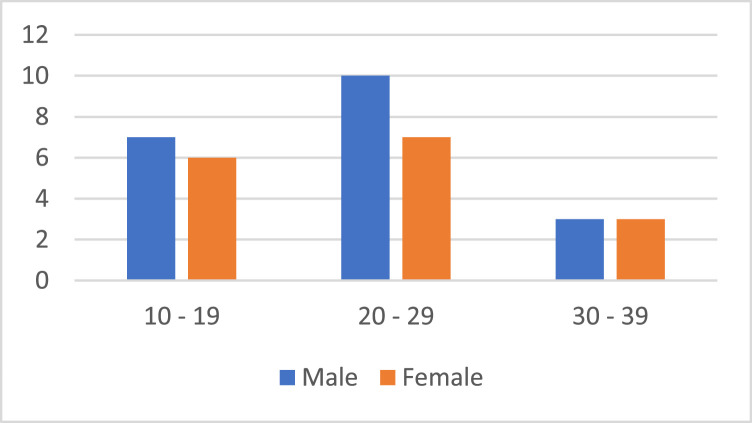
Distribution of participants by gender.

The ArYSL Version 2 dataset was developed to serve the research purpose on Arabic Sign Language through offering high-quality real-world sign gestures. Participants were employed using the convenience sampling strategy. A total of Thirty-five native Yemeni signers were selected based on their availability and willingness to participate in the study. While random sampling was not feasible due to logistical constraints and limited accessibility to registered Yemeni signers, efforts were made to ensure a diversity in age, gender, and regional dialects. Participants were recorded in naturalistic settings with uncontrolled background to simulate the methods of real-world application. This guaranteed wide-ranging linguistic and physiological representation. Recruitment was facilitated with the help of the local universities and deaf education institutions and community networks. The inclusion criteria included were being a native Yemeni Arabic signer, over 10 years old, literate, and capable to follow basic instructions. All participants provided informed, and irrevocable consent to participate as approved by the Institutional Review Board (IRB). The dataset includes sign language recordings from 35 native Arabic-speaking individuals from Yemen. The participants numbers were 20 male and 15 female aged between 10-40 years, with the average age of about 26 years. The distribution of gender is shown in Fig. 2.

**Fig. 3:**
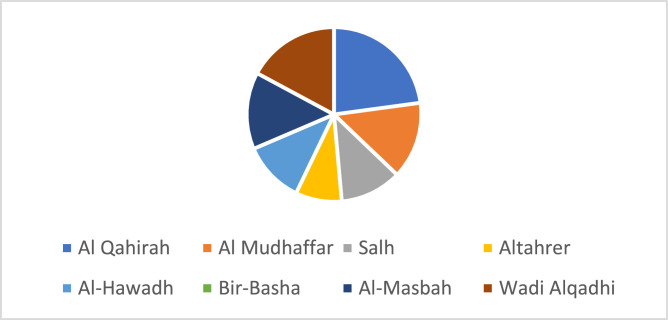
Distribution of participants by region.

Fig. 2 shows the proportion of 20 male and 15 female participants involved in the dataset collection. The geographic distribution of the study population was a combination of both urban and peri-urban environments, as shown in Fig. 3, and every participant was a resident on different districts in Taiz Governorate. The study included all individuals who were congenitally deaf or become deaf prior to three years of age and were fluent users of the Yemeni Sign Language (YSL). The demographic information was taken as voluntary to comply with ethical requirements.

Fig. 3, illustrates the geographic representation of participants from eight districts within the Taiz Governorate included Al Qahirah, Al Mudhaffar, Salh, Altahrer, Al-Hawadh, Bir-Basha, Al-Masbah, and Wadi Alqadhi.

### 4.3 Dataset image acquisition

**Fig. 4:**
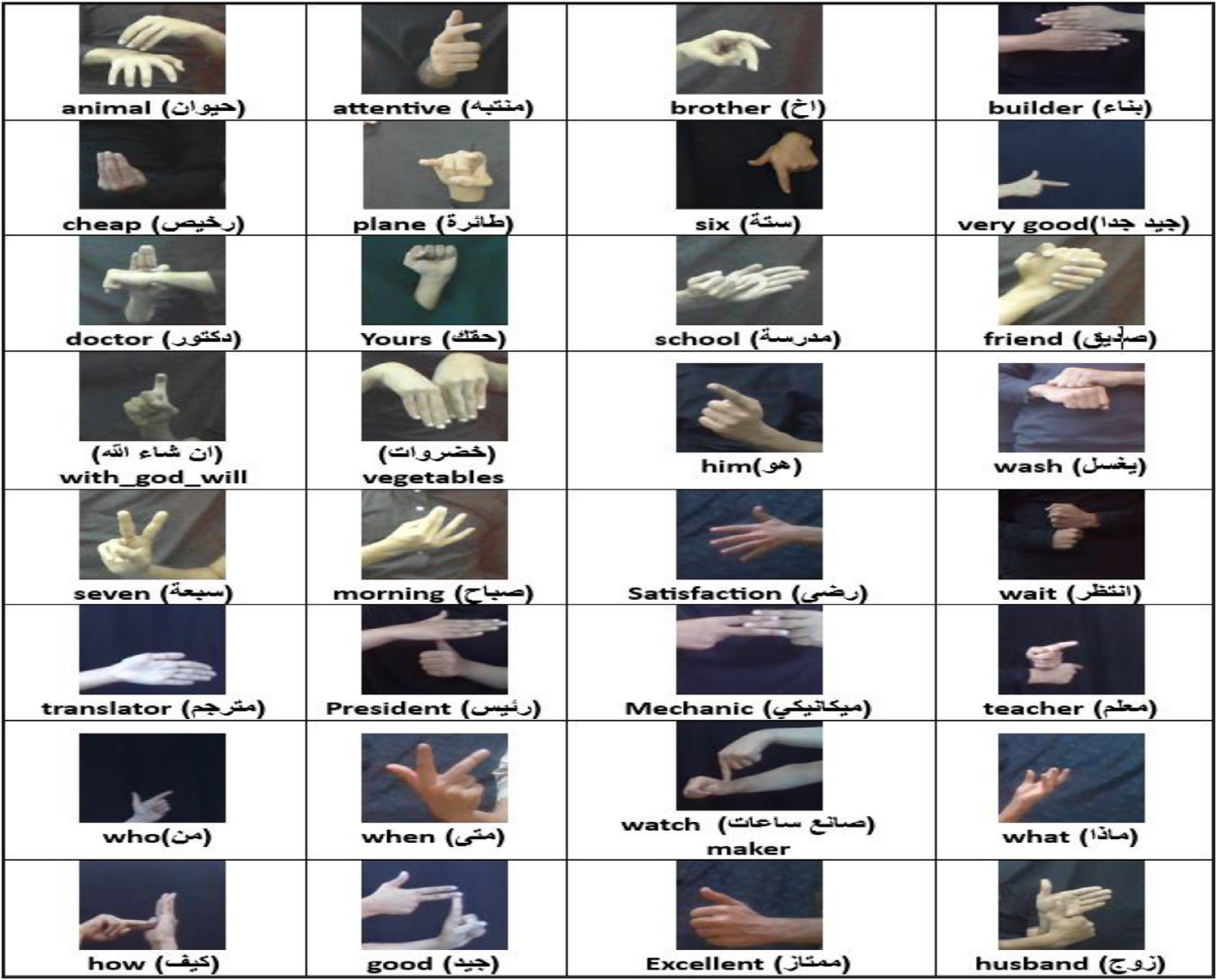
Sample sign images of the classes in the dataset.

The ArYSL Version 2 dataset is an extension of an earlier ArYSL Version 1, which was initially limited to 14 sign classes only. It incorporated 18 additional sign classes to expand the linguistic expressiveness of the dataset, resulting in a total of 32 commonly used signs. Classes were guided by the Unified Yemeni Sign Language Dictionary, and were selected based on their frequency and their importance on daily communication. All participants wore neutral clothing and maintained a neutral facial expression to emphasize hand gestures. Participants performed only isolated, static gestures to suit frame-based classification tasks. Each subject was instructed to produce 32 common signs using the Unified Yemeni Sign Language Dictionary. All signs were recorded as frozen, detached movements in indoor environment such as homes, offices, and classrooms in natural light, with varying backgrounds, without a green screen or studio lights to aid the simulation of real-world usage. Each participant was instructed to perform every sign a minimum of 10 to 15 times. The natural variability in the signing process was considered by capturing several numbers of image frames with every reiteration. Variations on hand orientation and signing pace were actively introduced to enhance the diversity and strength of the dataset. The process of image acquisition was administered at different distances between 3 and 9 meters, and camera angles were intentionally changed to provide greater visual diversity. The images were captured under various lighting conditions, including sunlight indoors and outdoors, during both day and night, with variations ranging from low to high illumination. There was no personally identifiable data gathered in line with approved ethics, including identity of signers and sign names. The sample images in Fig. 4 represent sign classes with different positions, lighting conditions and setting backgrounds.

### 4.4 Data preprocessing and organization

A custom-developed Python tool was used to manage the acquisition of sign images. Participants were requested to accomplish each sign multiple times to collect a diverse set of samples for each class. OpenCV library was utilized to record the streams capturing in frame-by-frame sequence with manual and automated snaps, and time-stamped saving of the images to the corresponding directories for each class. Raw images were filtered to eliminate duplicate or nearly identical images, poorly ghosted or blurry images, and incomplete gestures. Further pre-processing procedures were used to maintain consistency throughout the dataset by resizing to 224×224 pixels as well as histogram normalization of the contrast enhancement procedure. Table 2 displays the dataset summary for each class label and counted images.

**Table 2:** Summary the dataset for the multiple classes.

Class Label	Image Count	Class Label	Image Count
Animals	700	Mechanic	663
Attentive	700	Morning	700
Doctor	700	Plane	700
Brother	700	President	725
Builder	706	Satisfaction	874
Cheap	700	School	700
Excellent	894	Seven	700
Friend	700	Six	708
Good	788	Teacher	663
Him	700	Translator	970
How	572	Vegetables	700
Husband	713	Very Good	1,250
What	834	Wait	700
Wash	700	When	821
Watch Maker	1,248	Who	761
With God’s Will	555	Yours	700

A full preprocessing pipeline was created to ensure consistency in model training, while preserving the natural variability of human signs. Each sign class is placed in a specific directory named by the English transliteration of the Arabic sign name like (/doctor/, /how/, /very_good/). The dataset is organized in the form of a hierarchical folder structure, with each single file bearing a similar naming scheme: [class]_[index].jpg (e.g., doctor_001.jpg, doctor_002.jpg). Custom Python scripts were developed to perform the image acquisition, preprocessing, and quality control including:

• *captureSignImages.py*: Captures the sign images with the webcam and stores them into guided classes folders.
• *image_preprocessing.py*: Performs the normalization, noise reductions, and resizing into 224×224 pixels through bilinear interpolation.
• *quality_control.py*: Filters out the bad quality and blurry pictures by calculating the sharpness and redundancy of the images.
• *generate_metadata.py*: Incorporates the hierarchy of the directory structure of the dataset, providing labels to the folders as classes, and producing the two required metadata files.
• *sign_language_dataset_v2.csv*, where the names of image files are associated with their classes.
• *sign_language_dataset_v2.npy*, which consists of the image arrays, processed to be loaded in batches during training.

Laplacian variance criterion was adopted to test the image quality by detecting blurry images, so that any image with variance value of less than 100.0 was excluded. Only those images that are retained with detected hand regions satisfying a bounding box width and height of at least 60 pixels and detection confidence score of 0.75 or above threshold are applied to ensure enough hand visibility. The image contrast was enhanced by implementing the CLAHE (Contrast Limited Adaptive Histogram Equalization) algorithm set in the OpenCV library, with the parameters as clipLimit=2.0 and tileGridSize=(8,8). Additionally, perceptual hashing and histogram similarity with the similarity score of 95% or above were used to remove duplicated images. The validated images obtained from the pre-processed phase were stored in formats of .JPG or .PNG in their respective directories containing the classes, ensuring a well-organized file structure compatible for computer vision libraries.

### 4.5 Annotation and label verification

Two trained annotators manually labeled all images relying on a previously planned list of classes and labels according to the Unified Yemeni Sign Language Dictionary. The annotation process was conducted to achieve the linguistic consistency and semantic perfection within the ArYSL Version 2 dataset. Additional validations performed to enhance annotation quality through the following steps:

• Inter-annotator agreement was assessed where any labeling conflicts were reviewed and resolved by a third independent reviewer with expertise in Yemeni Sign Language.
• A Cross-verification audit was also performed on a random 15 percent subset of their dataset as a means of evaluating annotation consistency and accuracy. This subgroup was re-evaluated independently by both annotators and the third reviewer.

### 4.6 Dictionary construction for text-to-sign translation

A text-to-sign dictionary was developed in parallel with the image dataset to enable bidirectional Arabic sign language translation. This resource was derived from the selected sign classes and consists of 357 frequently used Arabic words. Each word was manually mapped with its corresponding sign class in the image dataset. Prior to integration, all text entries underwent a set of normalization procedures:

• Inclusion of Arabic word diacritics to standardize and map input word across Arabic dialects
• Harmonization of spelling variants, and synonyms for each word.
• UTF-8 encoding for multilingual NLP compatibility

The resulting mappings were compiled into a CSV file structured with four columns in the Arabic dictionary file. Table 3 presents a sample translation from the structured Arabic-to-English text-to-sign mapping dictionary included in the ArYSL Version 2 dataset. It contains the original Arabic class names, their English equivalents, and semantically related terms such as synonyms, sensitive expressions, and commonly observed or predicted spelling errors, and dialectal variation, pronunciation differences, and user input variability. The common misspellings are also significant in anchoring fuzzy string- matching techniques in NLP applications. The English versions are presented here for easy interpretation, where the complete dictionary has been optimized with processing the Arabic language, and it also reflects linguistic peculiarities that are not entirely captured in the English language.

**Table 3:** English translated samples for the ArYSL version 2 text-to-sign dictionary: class names, semantic synonyms, and common misspellings.

Please note that this table serves only as an illustrative English translation of selected entries from the original structured dictionary file in the ArYSL Version 2 dataset. It is intended to provide non-Arabic speakers with a general understanding for the semantic structure that used within the Arabic dictionary file. The original dictionary is structured specifically for the Arabic language, considering its unique linguistic, morphological, and semantic characteristics. Arabic language had a rich structure of synonyms, nuanced variations, and orthographic patterns such as semantic ambiguity, phonetic, and input variations leading to loss nuance when directly translated into English language.

The dictionary can be considered as an essential tool in downstream activities such as semantic word alignment in NLP pipelines, and real-time text-to-gesture translation. ArYSL Version 2 dataset provides more comprehensive framework for multimodal of Arabic sign language research by integrating the dictionary data with sign data images. This resource was developed to support the creation of bidirectional translation systems through the ability to convert existing forms of Arabic or spoken Arabic into appropriate translations of Arabic Sign Language. It holds significant value in Arabic–YSL translation systems for semantic parsing, word alignment, and gesture synthesis.

## Limitations

• All the signs in the dataset are static images and not included dynamic or video clips which are important for processing whole sentences and time data, which are critical for full sentence-level translation and temporal modeling.
• Partially balanced class distribution: Some class imbalance exists. So, to train or evaluate the deep learning models, special consideration should be for this influence.
• Limited diversity: more representative sampling across: additional regions within Yemen, age brackets, signing skill levels such as novice, intermediate, fluent. The broader range of signing words, styles, dialects, or physical characteristics not fully represent.
• Manual annotation: Despite careful review, the manual labeling process may contain minimal inconsistencies or noise due to human error.
• Acquisitions conditions were partially consistent for example, some participants used black backgrounds, which may affect the model generalization.
• Images were taken in an uncontrolled environment to introduce inconsistent images in lighting and resolution.
• Some sign classes have unequal image counts due to their semantic importance and filtering constraints, potentially impacting model fairness. This imbalance can be addressed through some techniques such as class reweighting, data augmentation, or stratified evaluation.
• The current dataset focuses on static, isolated signs, while future versions will incorporate dynamic sequences, facial expressions, and temporal data to enable sentence-level translation and multimodal sign analysis.

These important limitations are highlighted for researchers intended to adapt this dataset for training models, running performance tests or deploying solutions.

## Ethics Statement

The data collection protocol strictly adhered to ethical guidelines involving human participants. Informed consent was obtained from all subjects prior to image acquisition. The study complied with the Declaration of Helsinki and was approved by the relevant Institutional Review Board (IRB), under Protocol Number: TU-IRB-2024-017. All participants provided informed consent to ensure participant privacy. The dataset is intended solely for academic and research use. No data from social media or animal experiments were involved in this study. The authors confirm adherence to the ethical requirements of *Data in Brief* and declare no conflicts of interest regarding data collection.

## CRediT Author Statement

**Mogeeb A. A. Mosleh:** Supervision, Project Administration, Conceptualization, Methodology, Data Curation, Writing – Original Draft. **Rehab M.**: Software Development, Data Acquisition, Validation. **Ahmed M.**: Formal Analysis, Visualization, Writing – Review & Editing. **Abdu H.**: Writing – Review & Editing

**Code Availability**

The complete source code for image acquisition, preprocessing, and construction of the text-to-sign dictionary is publicly available via FigShare at: https://doi.org/10.6084/m9.figshare.26114395.v1. The code is released under an open-source license and is intended for academic and research use.

## Declaration of Generative AI and AI-assisted Technologies in the Writing Process

During the preparation of this work, the author(s) used OpenAI’s ChatGPT to assist with language refinement, grammar correction, and clarity improvements in the manuscript text. After using this tool, the author(s) carefully reviewed, verified, and edited the content to ensure accuracy, scientific integrity, and coherence, and take full responsibility for the content of the publication.

## Data Availability

FigShareArYSL: Arabic Yemeni Sign Language Dataset. (Original data).
